# Principal component analysis of phenotypic and breeding value data for semen traits in Egyptian buffalo bulls

**DOI:** 10.1007/s11250-024-03975-3

**Published:** 2024-04-22

**Authors:** Amin M S Amin, Mohamed M I Salem, Ayman F Ashour, Ayman G EL Nagar

**Affiliations:** 1 Animal Production Research Institute (APRI), Agricultural Research Center (ARC), Ministry of Agriculture and Land Reclamation, Dokki, Giza Egypt; 2 Department of Animal and Fish Production, Faculty of Agriculture, University of Alexandria, Alexandria, 21545 Egypt; 3 Department of Animal Production, Faculty of Agriculture at Moshtohor, Benha University, Benha, 13736 Egypt

**Keywords:** Egyptian buffalo, Semen quality, Principal components, Multivariate, Bayesian analysis

## Abstract

Buffalo bull semen traits are economically important traits that influence farm fertility and profitability. Genetic improvement of semen characteristics is an important detail of the genetic improvement. This study was conducted to assess the relationship between the breeding values as well as the phenotypic values for semen traits (VOL, MM, LS, AS and CONC) of the Egyptian buffalo bulls. A total of 7761 normal semen ejaculates were collected and characterized at ILMTC laboratory from 26 bulls from 2009 to 2019. For VOL, MM, LS, AS, and CONC, the actual means were 3.89 mL, 62.37%, 60.64%, 3.94%, and 0.67 × 109 sperm/mL, respectively. The prediction of breeding values for semen traits was estimated using a Bayesian procedure. The estimated standardized EBVs and phenotypic values were used in the principal component analysis (PCA). Of five PCs, one PC (PC1) had > 1 eigenvalues that was responsible for 87.19% of the total variation of SEBV, and two PCs had > 1 eigenvalues that were responsible for 59.61% and 21.35% of the total variation of the phenotypic values. Together, PC1 and PC2 accounted for 97.97% of the total variance of SEBV and 80.96% of the total variance of phenotypic values. A graphs of the first two components showed the traits separated into two different directions by group. This indicates each group was under similar genetic influence. Therefore, selection can be done separately for each group without influencing the other. Principal component analysis reduced variables to describe the key information in buffalo semen data.

## Introduction

In South America, Africa, Asia, and the Mediterranean, including Egypt, water buffaloes (Bubalus bubalis) are regarded the major dairy animal and the most important dairy species (Hernández-Castellano et al., 2019). Water buffaloes are Egypt’s primary source of milk, accounting for more than 45% of the country’s yearly milk production (FAOSTAT, 2020), and it is the world’s second-most-consumed milk (Freitas et al., 2020). Egypt has 3.4 million head of buffaloes, producing around 2,650,000 ton of milk (Hassanat et al., 2017). The number of Egyptian buffaloes has recently increased by 2.01%, making Egypt the sixth-largest buffalo-producing country in the world (FAOSTAT, 2020). Buffaloes are more adapted to the Egyptian hot climate conditions and more resistant to endemic diseases. Moreover, buffaloes outperform cattle in their capacity to adapt to hot, long productive life, humid, muddy, and marshy environments, allowing buffaloes to be economically viable in resilient environments compared with other animals (Vale, 2007). Buffalo is also regarded as a more environmentally friendly animal than other ruminants because of their reduced methane output. For example, buffalo create 157 g of methane per day/head, which is 58% less than that of cows (376 g per day/head) (Appuhamy et al., 2016). The Egyptian population increases rapidly, therefore demand on animals products such as milk and meat increases consequently. Increasing the number of animals to cover this increase in demand of animal products is limited due to high nutrition cost of animals in Egypt. Consequently, it is essential to genetically improve the productive and reproductive performance of livestock including buffalo. In contrast, buffaloes are characterized by low reproductive performance, extended calving intervals, long days open, late reproductive maturity and higher age at first calving (Amin et al., 2021). Therefore, buffaloes need national genetic improvement programs in order to increase its production and reproductive efficiency. This entails the need of accurately estimates of the genetic parameters for economic traits. Additionally, the poor conception rate of Egyptian buffalo cows is strongly related to the quality of the bulls’ semen. In order to boost the rate of conception in Egyptian buffaloes, it may be useful to identify the genetic influences on seminal traits. The heritability and genetic relationships between various sperm quality traits can also be used to predict how these variables would respond to genetic selection.

Principle components analysis (PCA) is a multivariate statistics that reduces the number of associated traits to a small number of independent variables while minimizing loss of the original data (Bolormaa et al., 2010). The eigenvalues of the variables are calculated and explain more variability than the following principle components (Meyer, 2007). The magnitude of the estimated breeding values (EBVs) of various traits in livestock species has been reduced and their relationships have been examined through the application of PCA in the genetic studies (Salem et al. 2021b; Valsalan et al. 2020). Moreover, the principle components might comprise traits with low heritability estimates that are rarely used in a direct selection process (Buzanskas et al., 2013). Therefore, after estimating the genetic parameters between the studied traits (Salem et al., 2023) and finding some results representing high and low genetic relationships between the traits, it was necessary to investigate PCA.

The studies concerning the estimation of principal components for phenotypic data and breeding values for semen traits in livestock animals, especially in buffalo, are scarce. So, the objectives of the present study were: (1) to estimate the breeding values (EBVs) for semen traits (ejaculate volume, sperm mass motility, live sperms percentage, abnormal sperms percentage and sperm concentration), and (2) to explore the relationship between the EBVs for these traits using PCA in order to create a PCA-based selection index that may be utilized to improve semen traits in Egyptian buffalo bulls.

## Materials and methods

### Ethical statement

Animal manipulations, the experimental procedures, laboratorial analyses and protocols were approved by the Ethical Committee of the Animal Production Research Institute (APRI). The study was conducted under the approval number: ARC-APRI-50-23.

### Animals and studied traits

Twenty-six Egyptian buffalo bulls were raised at the International Livestock Management Training Center (ILMTC) in Kafr El-Sheikh Governorate, Egypt. The bulls weighed between 350 and 400 kg. They provided a total of 7761 semen ejaculates over ten years, from 2009 to 2019. The bulls were fed a daily diet. This included 4 kg of rice straw, 3 kg of clover hay, and 4 kg of concentrate feed. They were fed according to guidelines from NRC (2001).

First, the scrotum size of the bulls was measured according to the method described by Pant et al. (2003); bulls that were 18–24 months old and had a crotch size greater than his 19 cm were used for insemination. Each bull’s semen was collected twice a week at 8 am. Collection was done through an artificial vagina kept at 42–45 °C. The semen was then quickly transported in a 37 °C water bath to the ILMTC laboratory for analysis.

### Microscopic evaluation of fresh semen

The analyzed semen characteristics included ejaculate volume (VOL), mass motility (MM), living sperm (LS), abnormal sperm (AS), and sperm concentration (CONC; 109 spermatozoa/mL). Ejaculate volume was precisely measured in milliliters to the closest 0.1 ml. Microscopic evaluation of mass motility, viability, and abnormalities in bull semen was performed twice every week. The percentage of individual mass motility demonstrated in this study is graded or leveled percentage based on the intensity of motility as described by (Evans and Maxwell, 1987).

Mass motility evaluation was observed using 10x magnification by a New Holland light microscope. Observation of spermatozoa viability was carried out by spermatozoa staining using 2% eosin dyes. Viable or living spermatozoa are characterized by a white head, while for dead spermatozoa the head is stained red or pink color (Toelihere, 1993). Spermatozoa abnormalities were done by observing Nigrosin stained spermatozoa and noting the type of abnormality. Semen concentration was checked using an evolution UV-Visible spectrophotometer (SD4, Minitube, Germany).

### Statistical analysis

#### Prediction of breeding values

The prediction of breeding values for the studied semen traits was performed through univariate repeatability animal model implemented in the THRGIBBS1F90 software (Tsuruta & Misztal, 2006). Model parameters were estimated using a Bayesian procedure, which was based on sample statistics from marginal posterior distributions created with a Gibbs sampling algorithm. The Gibbs sampler algorithm comprised 500,000 iterations, discarding the first 50,000. Afterwards, one sample in each 40 was saved and features of interest of the marginal posterior distributions were obtained using the POSTGIBBSF90 software (Misztal et al., 2021). The following model was used to analyze each semen trait separately:

1 $$\varvec{Y}=X\varvec{b}+Z\varvec{d}+ W\varvec{a}+L\varvec{p}\varvec{e}+\varvec{e}$$

where $Y$ = vector of the observed semen trait; $\varvec{b}$ = vector of the fixed effects of season of semen collection, year of semen collection and the age of buffalo bulls with an incidence matrix $X$; $\varvec{d}$ = vector of test-day effects with incidence matrix $Z$; $\varvec{a}$ = vector of random effects of the additive genetic effect with incidence matrix $W$; $\varvec{p}\varvec{e}$ = vector of random effects of the non-additive permanent environmental effect with incidence matrix $L$; and $\varvec{e}$ = vector of random residual effects. Residuals were normally distributed with mean 0 and variance ${I}_{n}{{\sigma }^{2}}_{e}$, where ${I}_{n}$ the identity matrix with an order equal to the number of records and ${{\sigma }^{2}}_{e}$is the residual variance. The vector of additive (animal) genetic effects was assumed to be normally distributed with mean 0 and variance ${{A\sigma }^{2}}_{a}$, where $A$is the numerator relationship matrix among animals in the pedigree file and ${{\sigma }^{2}}_{a}$ is the additive genetic variance. The vector of permanent environmental effect effects was assumed to be normally distributed with mean 0 and variance ${{{I}_{c}\sigma }^{2}}_{pe}$, where ${I}_{c}$is the identity matrix with an order equal to the number of buffalo bulls and ${{\sigma }^{2}}_{pe}$ is the permanent environmental variance.

#### Principal component analysis

In principal component analysis, the phenotypic data and estimated breeding values for the studied traits was used. To prevent the influence of the varied scales and magnitudes of the traits, the direct estimated breeding values obtained for the studied traits in this study were standardized for mean zero and unit variance. As detailed by Boligon et al. (2016), the standardization was carried out as follows:

2 $${Z}_{i}=\left({X}_{i}- \stackrel{-}{X}\right)/ {S}_{i}$$

where ${Z}_{i}$ is the standardized value of ${X}_{i}$ variable, $\stackrel{-}{X}$ is the mean of the i^th^ traits, and ${S}_{i}$ is the corresponding standard deviation. Then, using the FactoMineR tool in R software, the standardized EBVs (SEBVs) were used in the PCA with covariance matrix (Husson et al., 2013). According to Kaiser’s criterion, the PCs that explained the largest percentages of variation (i.e., > 1eigenvalues) were chosen in the current study. A principal component score (PCS) was created from each PC by combining the SEBVs of each trait and weighing them by their corresponding standardized score coefficient (SSC) (Buzanskas et al., 2013). As a result, the PC might be used to assess animals for multiple traits. The following formula was used to calculate the SSC of each SEBV in each PC:

3 $${SSC}_{ij}= \frac{{eigenvector}_{ij}}{{eigenvalue}_{j}}$$

where ${SSC}_{ij}$ is the SSC of SEBVs of the i^th^ trait in the j^th^ PC. The PCSs were computed in the following way:

4 $${PCS}_{j1}= {\sum }_{i=1}^{m}{SSC}_{ij}{SEBV}_{il}$$

where ${PCS}_{j1}$ is the principal component score for the l^th^ animal in the jth principal component, ${SSC}_{ij}$ is the SSC of SEBVs of the i^th^ trait in the j^th^ PC, and ${SEBV}_{il}$ is the standardized breeding value of the i^th^ trait for the l^th^ animal.

The following formula was used to compute the principal components scores (index values) for each animal in each principal component:

5 $$\begin{aligned}&{PCS}_{1}=\\&0.433 {EBV}_{VOL}+ 0.464 {EBV}_{MM}+ 0.453 {EBV}_{LS}+ \\&0.422 {EBV}_{AS}+ 0.463 {EBV}_{CONC}\end{aligned}$$

## Results and discussion

### Descriptive statistics

Table 1 shows the actual means, standard deviations (SD), and coefficients of variation (CV%) for the semen characteristics of buffalo bulls, including ejaculate volume (VOL), mass motility (MM), live sperm (LS), abnormal sperm (AS), and sperm concentration (CONC; 10^9^ spermatozoa/mL). For VOL, MM, LS, AS, and CONC, the overall means were 3.89 mL, 62.37%, 60.64%, 3.94%, and 0.67 × 10^9^ sperm/mL, respectively. These means fell within the range that had been reported by other research for dairy and buffalo bulls (Nazir et al., 1987; Söderquist et al., 1991; Ramadan, et al., 2009; Khattab et al., 2015 and 2022). According to Mahmoud et al. (2013), the overall averages for VOL, MM, and LS in Egyptian buffalo bulls were 2.9 mL, 70.9%, and 65.8%, respectively. Khattab et al. (2015) reported that actual means for VOL, MM, LS were 3.26 mL, 58.89% and 65.96%, respectively. VOL, LS, AS, and CONC were reported to be 2.6 mL, 67.3%, 18.4% and 526.28 × 10^6^ sperm/mL by Gabr and El Basuini (2018). Additionally, Rushdi et al. (2017) found that MM, LS, and AS were, respectively, 66.20%, 70.58%, and 15.15%. There are a number of possible explanations for the discrepancies between the current means of semen traits and those reported by various researchers working on different breeds of buffalo or dairy bulls, including individual differences in the bulls’ genetic makeup, age of the bull, nutrition of the bulls, season and year of collection, and other management and environmental conditions (Khattab et al., 2015 and 2022). For semen traits, the coefficients of variation (CV%) were modest and ranged from 25.17 to 46.57%. In the current study, the greatest CV% for ejaculate volume (46.57%) reflects the significant variation in semen volume amongst bulls. These estimations were consistent with those of Khattab et al. (2015), which reported that the CV% for semen traits in Egyptian buffalo bulls varied from 21.86 to 38.61%. Furthermore, Holstein dairy bulls had a CV% for VOL of 50%, according to Taylor et al. (1985).

**Table 1:** Summary statistics for semen traits in egyptian buffalo bulls

Trait^*^	Mean	SD	CV%	Min	Max
Ejaculate volume (mL)	3.89	1.81	46.57	0.50	11.50
Mass Motility (%)	62.37	15.85	25.41	12.00	94.00
Live sperm (%)	60.64	15.26	25.17	10.00	87.00
Abnormal sperm (%)	3.94	1.72	43.53	1.00	9.00
Concentration^†^	0.67	0.16	24.49	0.20	0.95

^*^Total number of records is 7761; ^†^Sperm concentration (10^9^ spermatozoa/mL)

### Principal component analysis

The variance components, genetic parameters and genetic and phenotypic correlations of VOL, MM, LS, AS, and CONC traits were previously presented by Salem et al. (2023) based on a subset of current ILMTC herd data. High and low heritability estimates along with low and high genetic correlations between these traits suggest that direct selection of some traits increases semen quality in Egyptian buffalo bulls which may be an efficient way to improve fertility. Therefore, the choice of PCA method is important for genetic improvement (Boligon et al., 2016), especially in the presence of traits with low heritability (e.g. 0.08 ± 0.07 for VOL and 0.04 ± 0.04 for AS) and low genetic correlations between traits (e.g. 0.17 ± 0.74 between VOL and MM and 0.22 ± 0.72 between VOL and LS) as shown in Salem et al. (2023).

The first principal component (PC1) accounted for 87.19% of the total variation in selective breeding values based on having an eigenvalue greater than one, as shown in Table 2. Additionally, two PCs had eigenvalue greater than 1 (Table 3) and explained 80.97% of the total phenotypic variation (59.61% for PC1 and 21.35 for PC2).

As shown in Table 4, the SEBVs for VOL, MM, LS, AS and CONC correlated with PC1. These results indicate that using animals higher in PCS1 calculated from Eq. 5 in selection programs will increase the semen production traits. Although using previous animals in selection programs will improve most of semen traits of current study, the rate of abnormal sperm will increase too.

As shown in Table 5, the phenotypic values of VOL, MM, LS and CONC correlated with PC1, while the phenotypic values of VOL and AS correlated with PC2. Together, PC1 and PC2 accounted for 80.96% of the total variation in phenotypic values. This indicates that the first two principal components were sufficient to explain most of the variation in phenotypic values for semen traits in Egyptian buffalos.

**Table 2:** Eigenvalues and variance proportions for the principal components (PCs) of the standardized breeding values for semen traits in egyptian buffalo bulls

PC	Eigenvalue	Variance %	Cumulative variance %
1	4.35	87.19	87.19
2	0.539	10.78	97.97
3	0.083	1.66	99.63
4	0.016	0.33	99.96
5	0.001	0.04	100.00

**Table 3:** Eigenvalues and variance proportions for the principal components (PCs) of the phenotypic values for semen traits in egyptian buffalo bulls

PC	Eigenvalue	Variance %	Cumulative variance %
1	2.98	59.61	59.61
2	1.06	21.35	80.97
3	0.881	17.63	98.61
4	0.059	1.19	99.80
5	0.009	0.190	100.00

**Table 4:** Correlation coefficients between the standardized estimated breeding values of semen traits in egyptian buffalo bulls and the first five principal components

PC	PC1	PC2	PC3	PC4	PC5
Ejaculate volume	0.904	0.385	-0.173	0.052	-0.003
Mass Motility	0.968	-0.205	-0.114	-0.083	0.014
Live sperm	0.945	-0.306	0.085	0.072	0.019
Abnormal sperm	0.881	0.436	0.177	-0.036	0003
Concentration	0.966	-0.253	0.032	-0.002	-0.034

**Table 5:** Correlation coefficients between phenotypic values of semen traits in egyptian buffalo bulls and the first five principal components

PC	PC1	PC2	PC3	PC4	PC5
Ejaculate volume	0.271	0.618	-0.737	0.001	0.001
Mass Motility	0.989	-0.074	0.0638	-0.068	0.074
Live sperm	0.986	-0.060	0.052	-0.127	-0.061
Abnormal sperm	0.043	0.819	0.571	0.0003	0.0002
Concentration	0.975	-0.071	0.061	0.197	-0.013

Some studies agreed or disagreed with the results obtained, but focused on other properties of the seeds. (Gilmore et al., 2021) in Holstein bulls showed the presence of two major principal components that contribute significantly to total variance, PC1 (60.6% of total variance) and PC2 (39.4% of total variance), but Aggarwal et al. (2007) and Dorado et al. (2010) reported that three main principal components influencing total variance.

The negative correlation coefficient between the phenotypic values ​​of MM and PC2 and the positive correlation coefficient between the phenotypic values ​​of AS and the same principal component (PC2) indicate that animals with a low proportion of motile sperm showed a high percentage of abnormal sperm. Additionally, positive correlation coefficients between SEBVs of VOL trait and PC2 was 0.385, while − 0.253 between SEBV of CONC trait and PC2, revealed that the bulls with high ejaculate volumes had low sperm concentration/ejaculate. Moreover, the positive and high correlation coefficients (0.385 and 0.436) of SEBV of VOL and AS traits and PC2 and the high and positive correlation coefficients (0.618 and 0.819) of the phenotypic values for the two traits and the same principal component (PC2), respectively, indicated that bulls with high ejaculate volumes had high percentages of abnormal sperms.

**Fig. 1:**
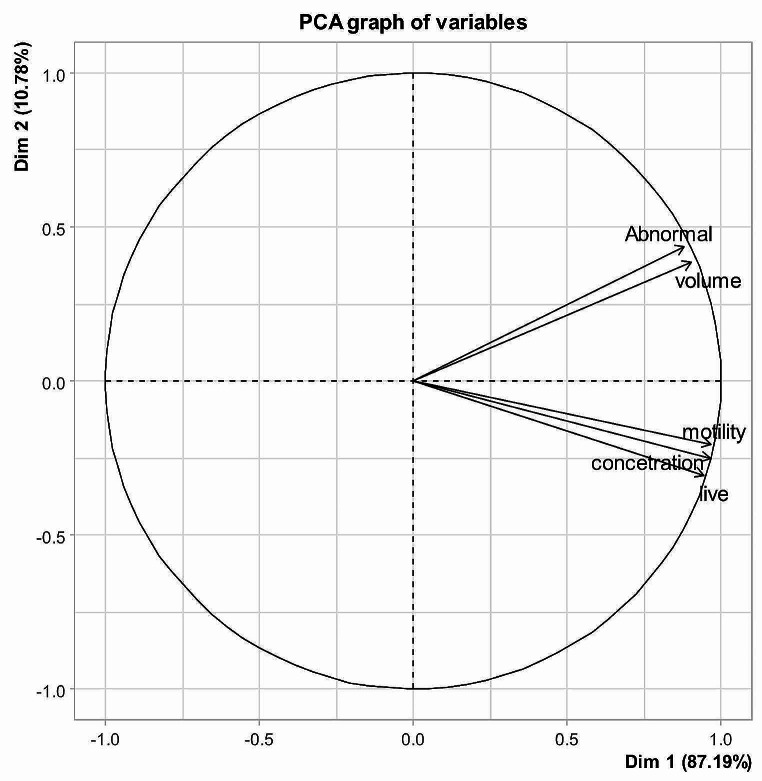
PC1 (Dim 1) vs. PC2 (Dim 2): Distribution of the estimated breeding values for the studied traits in each of the first two principal components. Volume (Ejaculate volume), motility (Mass Motility), live (Live sperm), abnormal (Abnormal sperm), concentration (sperm concentration)

**Fig. 2:**
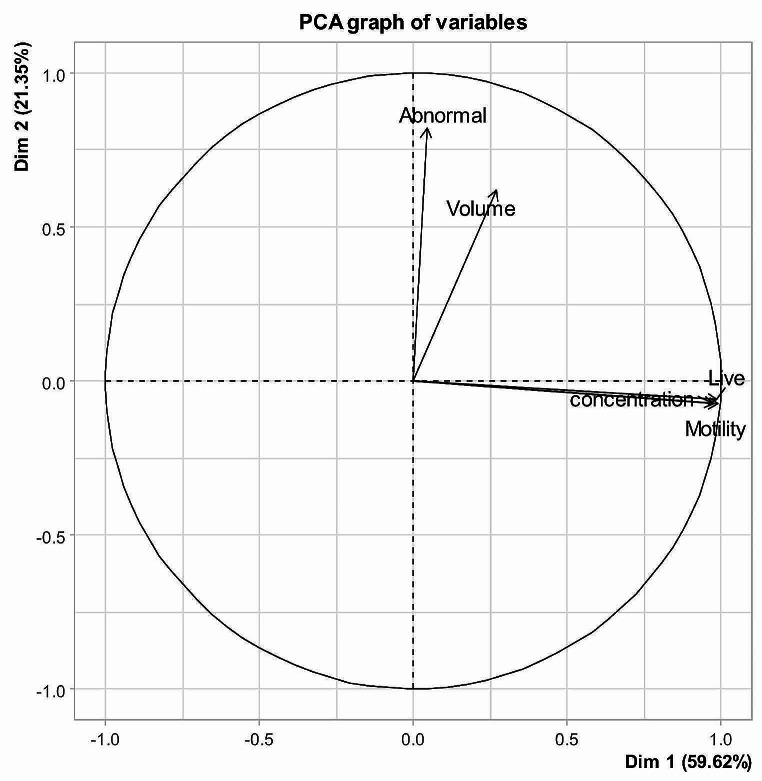
PC1 (Dim 1) vs. PC2 (Dim 2): Distribution of the phenotypic values for the studied traits in each of the first two principal components. Volume (Ejaculate volume), motility (Mass Motility), live (Live sperm), abnormal (Abnormal sperm), concentration (sperm concentration)

Moreover, the two-dimensional graph of PC1 vs. PC2 obtained from SEBV and phenotypic data (Figs. 1 and 2, respectively) presented that the investigated traits were distributed in two different directions according to their groups (VOL and AS in group 1, and MM, LS and CONC in the other group), indicating that each group under the same genetic influence. Thus, selection for each group could be done separately away from the other groups (Buzanskas et al., 2013). These results are consistent with Tables 4 and 5, where MM, LS, and CONC are strongly associated with PC1, and VOL and AS are more closely associated with PC2. These results are consistent with those of Salem et al. (2023), where the results of genetic correlation is highest between the traits abnormality and the same is true for the traits Motility, live and Concentrate. The prior correlation coefficients between the various PC characteristics represented the complex interactions between these seminal traits as well as the genetic basis of the quantitative traits, which were controlled by a number of genes, each of which contributed differently to each trait.

Few studies have described the relationship between principal components and their semen traits in livestock. Aggarwal et al. (2007) was measured various five biometry of the sperm head (Maximum Length, Maximum width, Area, Perimeter and Maximum width/maximum length), and Biometry of the sperm tail (Length of midpiece, Width of midpiece and Length of tail) of frozen–thawed sperm from eight breeds of Indian buffaloes. Multivariate analysis PCA of eight sperm morphometric endpoints in eight breeds revealed that the proportion of variation explained by the first three principal components was 77.08, 17.50, and 3.29, respectively. In pig, the principal components of the sperm kinetic parameters at a video recording time of 2 s produced three principal components with a total explained variance of 92.0% (Valverde et al., 2019).

The relationship between Computer-assisted semen analysis (CASA) studies and principal component analysis determinations of semen properties provides several different pieces of information. Gilmore et al. (2021) were studied to determine the total antioxidant capacity of sperm as well as the number and cell characteristics of spermatozoa in Holstein bulls with good freezing tolerance and Holstein bulls with poor cold tolerance. Sperm dynamics after thawing were assessed using CASA. The total variance in sperm population characteristics is mainly explained by two main principal components: PC1 (60.6% of the total variance) and PC2 (39.4% of the total variance). Factor pattern analysis showed a strong correlation between PC1 and viable cell number, whereas PC2 was correlated with total motility. In another study, three principal components with eigenvalues greater than 1 were identified, accounting for 88.59% of the variance. Considering the results of CASA parameters, the PC1 is related to fast and linear movements, the PC2 is related to fast and irregular movements including lateral displacement of the wide head, and the PC3 is related to fast and irregular movements, including lateral displacement of the wide head. These results of this study indicate that CASA-derived sperm characteristics can be reduced to three major components, which account for more than 88% of the total variance in total motility, and sperm concentration shows that each individual’s sperm has different movement patterns (Dorado et al., 2010).

Molecular genetics provides an alternative view of the principal components based on the detection of QTLs. Haplotypes, eigenvectors, and phenotypes of 794 Swiss Brown Swiss bulls used to detect QTL for bovine chromosome no. 6 (Hiltpold et al., 2020). The archive includes graded genotypes (haplotype_6) at 28,872 SNPs (marker_6) on chromosome 6, the top 20 principal components, and the average sperm motility of 794 Brown Swiss bulls.

A number of studies have focused on other traits than semen traits, demonstrating the value of using contrast agents and their impact on genetic improvement. In this regard, Oliveira et al. (2014) assessed seven productive and two reproductive variables in Brazilian buffaloes, concluded that a reduced rank model with three or four PCs was sufficient to account for the majority of the additive genetic variance across all traits. Moreover, Agudelo-gómez et al. (2015) demonstrated that there were three PCs accounted for 65.87% of the variation in breeding value for growth traits in Colombian buffaloes. They added that PC1 was more strongly connected with breeding values of growth traits, PC2 with maternal genetic component traits, and PC3 with breeding values of milk yield traits. Furthermore, Salem et al. (2021b) conducting a principal analysis for birth weight, milk production and reproductive traits for the Egyptian buffalo; revealed that four of the eight principal components had more than one eigenvalue, accounting for 70.37% of total variance. They added that for PC1, PC2, PC3, and PC4, the variance that could be explained were 25.71%, 18.20%, 13.28%, and 13.18%, respectively. In Brazilian Canchim beef cattle, the standardized EBV for body weight at 420 days was strongly correlated with the second principal component, in contrast to the substantial relationship with the first principal component shown by the standardized EBVs for age at first calving, age at second calving, and calving interval (Buzanskas et al., 2013). Boligon et al. (2016) showed that three PCs accounted for 79% of the total breeding value variance of beef cattle’s growth and reproductive traits and noted that PC1 associated with the overall average performance of growth and reproduction parameters, but PC2 distinguished animals with higher or lower growth and PC3 distinguished animals with various. Valsalan et al. (2020) conducted principal component analysis for growth traits in Indian Malabari goats, found that four of the twelve principal components were accounted for 67.78% of the total variance; the first component explained 28.02% of the total variance.

## Conclusion

Observed breeding values with strong correlation coefficient to the first principal component than those with strong correlation coefficient to the second principal component. PC1 can be considered a genetic index of semen traits in the Egyptian buffalo bulls, because it favors animals genetically superior for EBV_VOL_, EBV_MM_, EBV_LS_, EBV_AS_ and EBV_CONC_. One PC was produced by the PCA used, which effectively reduced the dimensionality of the Egyptian buffalo data and captured 87.19% of the EBV variance for semen Traits. Two PCs were captured 80.96% of the variation in the phenotypic values for these traits. Animals with greater PC1 thus meet the breeding goals of farmers generating high-quality semen and would be more advantageous for Egyptian buffalo selection programmes. Additionally, kinematic, morphometric, morphological, or DNA integrity testing can be applied to characterize and understand sperm reproductive biology. Further studies should be devoted to evaluating the biological basis in terms of sperm competition, female migration along the route, environmental influences, and ultimately the determination of fertility.

## Data Availability

Available upon request.
